# Multicolour Fluorescence-Detection Size-Exclusion Chromatography for Structural Genomics of Membrane Multiprotein Complexes

**DOI:** 10.1371/journal.pone.0067112

**Published:** 2013-06-25

**Authors:** David Parcej, Renate Guntrum, Sabine Schmidt, Andreas Hinz, Robert Tampé

**Affiliations:** 1 Institute of Biochemistry, Biocenter, Goethe-University Frankfurt, Frankfurt am Main, Germany; 2 Cluster of Excellence – Macromolecular Complexes, Goethe-University Frankfurt, Frankfurt am Main, Germany; University of Technology Sydney, Australia

## Abstract

Many interesting and important membrane proteins are hetero-oligomeric. However, besides naturally abundant examples, the structures of relatively few such complexes are known. Partly, this is due to difficulties in expression, stoichiometric assembly, and in the evaluation of their stability prior to crystallization trials. Here we describe a new approach, which allows rapid assessment of protein complex quality, assembly and stoichiometry, simplifying the search for conditions conducive to long-term stability and crystallization. Multicolour fluorescence size-exclusion chromatography (MC-FSEC) is used to enable tracking of individual subunits through expression, solubilization and purification steps. We show how the method has been applied to the heterodimeric transporter associated with antigen processing (TAP) and demonstrate how it may be extended in order to analyse membrane multisubunit assemblies.

## Introduction

In the post-genomic era, the high-resolution structure determination of higher eukaryotic integral membrane proteins has lagged behind [1]–[3]. The reasons for this are well known. Firstly, rich natural sources of these proteins are rare and membrane proteins are often difficult to over-express in functional form. Even if they can be well expressed, they may assemble in complexes with ill-defined stoichiometries. Moreover, once extracted from the membrane prior to purification and crystallization, membrane proteins are frequently unstable, becoming aggregated or losing function. This problem is generally addressed by varying the conditions in which the protein is brought and kept in solution. Most often, numerous detergents and additives such as lipids, ligands, fusion approaches and conformation specific antibodies must be tested, a lengthy and tedious undertaking with no guarantee of success [4]–[8]. In addition, the protein itself may be modified, often randomly or in a systematic manner (see review [9]).

Recently, this lengthy pre-crystallization phase has been shortened by using fusions with the green-fluorescent protein (GFP) [10], [11]. When fused to the C terminus of a protein, it appears that the GFP will only fold into the correct fluorescent form if the preceding target protein is likewise correctly folded [12], [13]. This allows for detection of levels of expression in host cells (for example [14]). Most importantly, when coupled to size-exclusion chromatography, the GFP may be used to assess the oligomeric state of the fusion protein even in crude detergent extracts [15]. This method has been dubbed FSEC and has recently been extended to allow determination of thermal stability (FSEC-TS) [16]. However, to our knowledge this method has thus far only been used for proteins coded by a single gene, either as a monomer or assembled into homo-oligomers. But many higher eukaryotic proteins are hetero-oligomeric complexes and FSEC as used is not suitable.

Lately, we have shown that it is possible to express the human ATP-binding cassette (ABC) transporter associated with antigen processing (TAP) in *Pichia pastoris* and to purify it in a functional state [17]. TAP forms an obligate heterodimeric complex, which performs the vital function in the translocation of proteasomal degradation products from the cytoplasm into the endoplasmic reticulum, where they are loaded onto major histocompatibility complex (MHC) class I molecules for surface presentation to cytotoxic T-lymphocytes. Thus, TAP is a key component of the adaptive immune response to pathogens and cancer cells. Because of its crucial function, TAP is targeted by a number of viruses (for review see [18]). To extend further our understanding of the mechanism of transport and substrate selection by TAP, we now wish to determine its high-resolution structure by X-ray crystallography.

In order to attack the pre-crystallization phase mentioned above, we have developed an extension of the FSEC technique, which we call multicolour fluorescence (MC)-FSEC. The key requirement of MC-FSEC is the ability to detect multiple subunits of membrane protein complexes, such as TAP1 and TAP2, simultaneously and to analyse their behaviour in numerous conditions. In order to achieve this, TAP1 was fused at the C terminus with an enhanced YFP, monomeric Venus (mVenus), followed by a His_10_-tag for purification. TAP2 on the other hand was constructed with monomeric Cerulean (mCeruelan), a brighter variant of CFP at the C terminus plus a strepII-tag. As shown in Figure S1, there is some overlap between the emission spectrum of mCerulean and the excitation spectra of mVenus and this property is often used for Förster resonance electron transfer (FRET) studies. Because in our constructs, the fluorescent proteins are linked to the target subunit *via* a flexible linker, FRET effects are negligible and by appropriate selection of excitation and emission wavelengths on a dual detector system, separate detection of both signals is simple.

The use of the different purification tags allowed us to develop an orthogonal purification strategy that can be used to isolate hetero-oligomeric complexes with defined stoichiometry. During this process separate monitoring of the two subunits on size exclusion chromatography (*i.e.* MC-FSEC) allows rapid assessment of the correct assembly and stoichiometry under the conditions tested. In this report we illustrate the utility of this method that may be suitable for other hetero-oligomeric membrane protein complexes.

## Materials and Methods

### Construction of mVenus and mCerulean*Pichia* Expression Vectors

**Figure 1:**
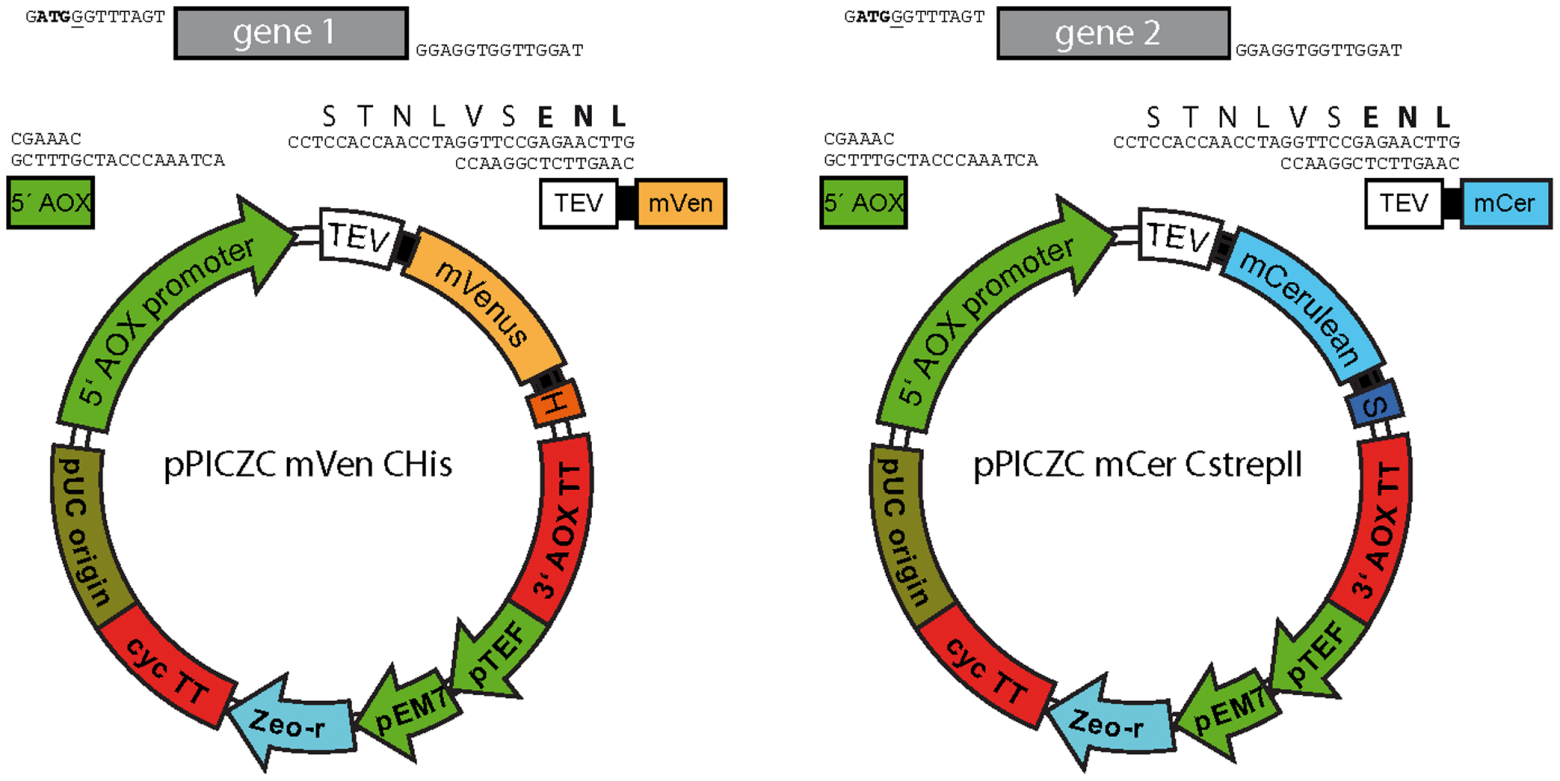
Cloning different subunits for*P.* *pastoris* expression of multisubunit membrane complex in-frame with fluorescent proteins. Shown are the vectors containing mVenus and His_10_-tag (left) and mCerulean including a strepII-tag (right) used to clone different subunits for co-expression in *P. pastoris.* Salient features are: H, His_10_-tag; S, strepII-tag; TEV, TEV protease cleavage sequence; mVenus and mCerulean, the fluorescent proteins. Upper parts show a close-up of the region between the AOX1 promoter (green) and the TEV sequence (white, start of the TEV sequence is given in bold letters) after digestion with BsmBI and T4 DNA polymerase. Also shown is a theoretical PCR product (any gene of interest) after T4 DNA polymerase treatment displaying ends complementary to the vector.

The vector pPICZC (Invitrogen) was modified to produce two vectors differing only in the coded fluorescent proteins and purification tags (mVenus with a His_10_-tag and mCerulean with a StrepII-tag). This was accomplished by inserting fluorescent protein DNA into the vector pPICZC and by introduction of oligonucleotides encoding the purification tags and TEV cleavage site (Figure 1). To allow rapid insertion of target open-reading frames, an oligonucleotide containing two BsmBI sites was introduced immediately after the end of the AOX1 promoter sequence. Cleavage by this enzyme followed by digestion with T4 DNA polymerase in the presence of excess dCTP produced the overhangs shown in Figure 1. To clone TAP variants, their genes were amplified by PCR using primers containing the following overhang: 5′: G**ATG**GGTTTAGTC where the start codon is shown in bold. 3′: TAGGTTGGTGGA*GGC* (here the reading frame is shown in italics). Digestion of the product with the same enzyme except with excess dGTP in the reaction will generate the overhangs indicated in Figure 1. The vector and insert may then be mixed and used directly to transform competent *E. coli* cells. This procedure is ligation-independent cloning (LIC) [19].

### Transformation of*Pichia pastoris*

SMD1163 protease-deficient cells were previously transformed with the empty pAO815 vector (Invitrogen), using the protocol described below in order to produce a His^+^ strain for easy growth in minimal media. Plasmid DNA (50 µg) was digested using the MssI site in the 5′AOX promoter region. After purification using the Qiagen Qiaquick PCR purification kit (with elution in water), linearized plasmids for gene 1 and 2 (30 µg each) were mixed and concentrated in a Speedvac concentrator to 10–20 µl. *Pichia* cells were made competent using the described method [32]. Briefly, cells were grown overnight in YPD medium (1% yeast extract, 2% peptone, 2% dextrose; 10 ml per transformation reaction) until the OD_600_ was 1.2 to 1.3. Cells were centrifuged for 5 min at 1,500×g and re-suspended in half the original growth volume of 10 mM Tris/HCl, pH 7.5, 100 mM lithium acetate, 600 mM sorbitol, 10 mM dithiothreitol (DTT). After 30 min at room temperature, the suspension was once more centrifuged, transferred to a 2 ml centrifuge tube, and washed three times with ice-cold 1 M sorbitol. The final pellet was then suspended in 1 ml ice-cold 1 M sorbitol and the OD_600_ of a diluted aliquot measured. Assuming 1 OD_600_ unit to be equivalent to 5×10^7^ cells/ml, 5×10^8^ cells (total volume made to 0.1 ml) were incubated with the linearized DNA for 5 min on ice. They were then transferred to an ice-cold electroporation cuvette (2 mm gap) and electroporated with a BioRad Genepulser II at 2.5 kV, 25 µF and 400 Ohms. Immediately, 0.65 ml of sorbitol (1 M) was added and the cells transferred to a 15 ml conical tube, which was capped and allowed to stand 2 h at 30°C. An equal volume of YPD was then added and the tube shaken at 30°C for a further 2 h. The suspension was then distributed between YPDS plates (1% yeast extract, 2% peptone, 2% dextrose, 1 M sorbitol, 15 g/l agar) containing 2, 3, or 4 mg/ml Zeocin (two plates each). Colonies became visible after three days at 30°C.

### Screening of*Pichia pastoris* Colonies for Expression

Colonies from transformation plates were picked and used to inoculate 2 ml of MGY (3.4 g/l yeast nitrogen base, 10 g/l ammonium sulphate, 0.4 mg/l biotin, 1% (w/v) glycerol) in 10 ml deep-well plates. After one day shaking at 30°C, aliquots were removed and used to inoculate a second deep-well plate containing 2 ml of MGY. These cells were then grown until an OD_600_ of 5.0 was reached. Cells were then centrifuged, the pellet suspended for induction in 10 ml of MM medium (3.4 g/l yeast nitrogen base, 10 g/l ammonium sulphate, 0.4 mg/l biotin, 0.5% methanol), and transferred to 50 ml conical tubes. These were shaken for 24 h at 30°C and the cells harvested by centrifugation at 1,500×g for 5 min. This pellet was suspended in 500 µl of breaking buffer (50 mM Na-phosphate, pH 7.4, 1 mM EDTA, 100 mM amino-hexanoic acid, 5% (w/v) glycerol, 2.5 mM benzamidine, 0.1 mg/ml soybean trypsin inhibitor, 1 mM PMSF) and transferred to a 2 ml centrifuge tube. An equal volume of ice-cold glass beads (0.5 mm diameter) was added and cells broken using a FastPrep-24 (MP Biomedicals) for 2×1 min at 6.5 m/s. Extra breaking buffer (1 ml) was added, the glass beads resuspended by shaking and the mixture centrifuged at 2,000×g for 5 min to remove cellular debris. The supernatants were then removed and centrifuged at 50,000×g for 30 min to collect the membranes. Pellets were frozen or used immediately.

For solubilization, pellets were suspended in 75 µl of water and an equal volume of twice-concentrated solubilization buffer (100 mM Hepes, pH 7.4, 400 mM NaCl, 100 mM KCl, 2 mM PMSF, 5 mM benzamidine) containing 2% (w/w) *fos*-choline-12 (FC-12). After mixing 1 h at 4°C, the samples were centrifuged 100,000×g for 1 h. Supernatants were filtered through 0.2 µm SpinX centrifuge filters (Corning Life Sciences) and 50 µl of the filtrate used for MC-FSEC.

### Scale-up of Expression

Colonies, which were identified using the expression screen to produce both subunits in reasonable amounts, were used to seed 50 ml of MGY in a 250 ml Erlenmeyer flask. After overnight culture at 30°C, this in turn was used to inoculate 800 ml of MGY in a 2 l Erlenmeyer flask. When the culture reached an OD_600_ of 5 to 6, it was centrifuged at 1,500×g for 5 min and suspended in 2 l of MM, which was put into a 5 l baffled Erlenmeyer flask. After 24 h at 30°C, cells were collected by centrifugation and frozen until processed to produce membranes using the FastPrep-24 as above but scaled up to use 15 ml conical tubes instead of 2 ml centrifuge tubes. Typically, 200 to 300 mg membrane protein is obtained.

### High-throughput Orthogonal Purification

This procedure utilized a step of immobilized metal affinity chromatography (IMAC) in batch mode to isolate complexes containing the subunit harbouring the His_10_-tag followed by batch adsorption and elution from streptactin-Sepharose to further isolate complexes that also contained the strepII-tag. Membranes were solubilized at 5 mg/ml for 1 h in solubilization buffer containing 2% (w/v) of the detergent to be tested. The final volume was 1.5 ml. After centrifugation at 100,000×g for 1 h, the supernatant, which we refer to as crude extract, was added to 0.1 ml of Ni-NTA beads (His-select, Sigma), which had been washed with IMAC wash buffer (40 mM imidazole/HCl, pH 7.5, 200 mM NaCl, 50 mM KCl, 0.1 mM PMSF, 0.25 mM benzamidine, plus 2–10× the CMC of the detergent). After 2 h mixing at 4°C, the beads were washed by centrifugation at 100×g for 1 min, the supernatant (IMAC flow-through) removed and 1 ml of IMAC wash buffer added. The beads were mixed for 3 min to allow complete equilibration and the process repeated three times. To elute the protein, the matrix was suspended in 0.5 ml elution buffer (wash buffer but with 150 mM imidazole) followed by 30 min mixing at 4°C and centrifugation. The supernatant (IMAC eluate) was added to 50 µl streptactin-Sepharose (IBA, Goettingen, Germany) and mixed at 4°C for 1 h before collection of the strep flow-through by centrifugation and washing with IMAC wash buffer as above. Elution was accomplished by incubating the matrix for 45 min with 0.25 ml IMAC wash buffer containing 5 mM desthiobiotin. All procedures were conducted on ice or in a cold-room (4–8°C). Samples were taken at each stage of the purification and analysed by MC-FSEC. The eluate was analysed by SDS-PAGE (Coomassie stained) and in-gel fluorescence (Typhoon, GE Healthcare).

### Binding of Peptide and Viral Inhibitor to TAP

His_10_-ICP47 (S81C) was expressed in *E. coli* BL21(DE3)pLysS and purified as described [33]. The antigenic epitope RRYCKSTEL derived from human histone H3.3 was synthesized by Fmoc solid-phase chemistry. Single cysteine ICP47 and peptide were labelled with maleimido-Atto565 (ATTO-TEC GmbH, Siegen, Germany) and purified by reverse-phase C_18_-HPLC as described previously [34]. The identity of the fluorescently labelled products was verified by mass spectrometry. To initiate binding Atto565 labelled peptide or ICP47 were added at a final concentration of 2 µM or 1 µM respectively to TAP (1 µM) diluted in IMAC wash buffer and allowed to incubate for 20 min at 4°C before MC-FSEC. To assess non-specific binding, a 100-fold molar excess of unlabelled ligand was included in the binding reaction.

### MC-FSEC Analysis

An Agilent 1200 series high-performance liquid chromatography (HPLC) system kept in a cold-room was used. The system was equipped with an auto-sampler capable of high-throughput using 96-well microtiter plates. A Shodex semi-micro KW404-4F (4.6×300 mm) column was used for SEC analysis. Running buffer comprised 20 mM Tris/HCl, pH 7.2, 300 mM NaCl, 50 mM KCl, with 2–10× the CMC of detergent and the column temperature was controlled at 10°C except for binding experiments where 10% (w/v) glycerol was added to the running buffer and the column equilibrated at 0°C to reduce ligand dissociation during chromatography [34]. Samples were pre-filtered through 0.2 µM SpinX centrifuge filters and pipetted into a 96-well plate for injection. Run time was 20 min at 0.3 ml/min. Data was analysed in GraphPad Prism. When the two FLDs were used for mVenus and mCerulean, the settings were the following: FLD1: excitation/emission 435/470 nm, gain 12; FLD2: excitation/emission 515/535 nm, detector gain 10. These settings were confirmed to give equal fluorescence intensity using purified mVenus and mCerulean proteins (Figure S1). In the three-colour experiments FLD1* setting was for single emission detection at 520 nm with dual excitation at 430 nm (for mCerulean) and 510 nm for mVenus with a gain of 12. This allowed the second detector FLD2* to be used for detection of the red fluorophore (in this case Atto565 excitation/emission at 563/589 nm, gain 14).

## Results and Discussion

### Vectors for Subunit Fusions

Vectors for cloning of subunits of a complex fused to fluorescent proteins were constructed using the expression vector pPICZC as a starting point. One of the vectors contains an mVenus encoding sequence followed by a His_10_-tag and was used in these studies for cloning of TAP1 (Figure 1). A similar plasmid backbone was used for the TAP2 construct, except for the replacement of mVenus by mCerulean and the His_10_-tag by a strepII-tag. To facilitate rapid cloning of TAP variants from different species, a sequence allowing ligation-independent cloning (LIC) [19] was inserted after the strong inducible AOX1 promoter, which enables the use of high-throughput cloning platforms. We chose to use C-terminal fusions as reporters of correct folding [12], [13] and because the N termini of the subunits, particularly TAP2, are very close to the membrane [20], [21] and insertion of a large fusion protein at this position may interfere with folding and/or assembly of the TAP heterodimer. In contrast, we have found C-terminal fusions to have no deleterious impact on TAP expression levels or function [22], [23]. For other complexes it may be preferable to place tags on the N-terminus (or perhaps the best position is unknown). This would require a simple shuffling of the elements on the vectors illustrated. However, in this case, we would caution against relying on fluorescence alone as indicative of production of correctly folded fusion protein because it may arise from either truncated fusion protein or even folded fluorescent protein followed by an incorrectly folded fusion partner.

Both of our vectors contain the recognition site of the TEV protease, which allows easy removal of the fluorescent proteins and purification tags. In addition, flexible linker sequences (encoding GGGS) have been placed between the TEV cleavage site and the fluorescent protein and also before the purification tags. These linkers serve to ensure efficient cleavage by TEV and optimal association with affinity matrices.

### Expression Levels of Individual Subunits Assessed by Multicolour FSEC

**Figure 2:**
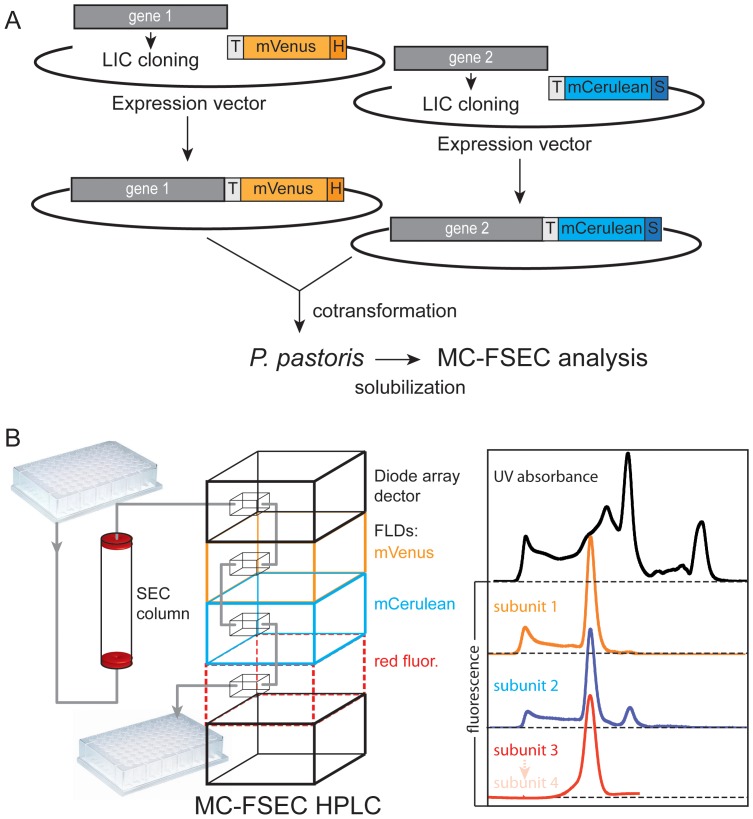
Workflow leading up to multicolour FSEC. (A) After LIC cloning into expression plasmids, genes encoding different subunits of a multisubunit complex are ready for co-transformation into *P. pastoris*. Salient features are: H, His_10_-tag; S, strepII-tag; TEV, TEV protease cleavage sequence; mVenus and mCerulean, the fluorescent proteins. After expression the complexes are analysed by MC-FSEC. (B) Scheme of the MC-FSEC setup. The chromatography system consists of an auto-injector, pump and column, followed by two fluorescence detectors (FLDs) and a diode-array detector for measuring absorbance.

Initially, we hoped to use in-cell fluorescence to measure the amount of each subunit produced by *Pichia pastoris*. We chose to co-transform with plasmids encoding TAP1 and TAP2 because expression levels may not reflect gene-copy number and producing individual constructs was simpler (Figure 2A). However, this meant it was necessary to analyse multiple colonies in order to find one with high and equal expression of both subunits. Unfortunately, although mVenus fluorescence could be readily detected, we were unable to assess expression levels of the second subunit in yeast cells by in-cell fluorescence, because of high background in the range of mCerulean emission. This does not appear to be a problem for fluorescence-detection in mammalian cells, but for *Pichia* we decided to exploit a normally problematic property of the TAP complex: its tendency to split into subunits in the presence of the harsh detergent *fos*-choline-12 [24], while still remaining in solution. Using this method, it was simple to select colonies producing equal amounts of the subunits (see below).

The multicolour fluorescence MC-FSEC system is schematically illustrated in Figure 2B and is a high-performance liquid chromatography system equipped with an auto-sampler for high-throughput and two fluorescence detectors in tandem as well as a detector for measuring absorbance. We have found that it is possible to detect both fluorescent subunits with a single fluorescence detector that allows either multiple excitation or emission wavelengths (Figure S1). However, for highest sensitivity and parallel monitoring of assembly of a third component of multiprotein complexes, we prefer the dual detector system.

**Figure 3:**
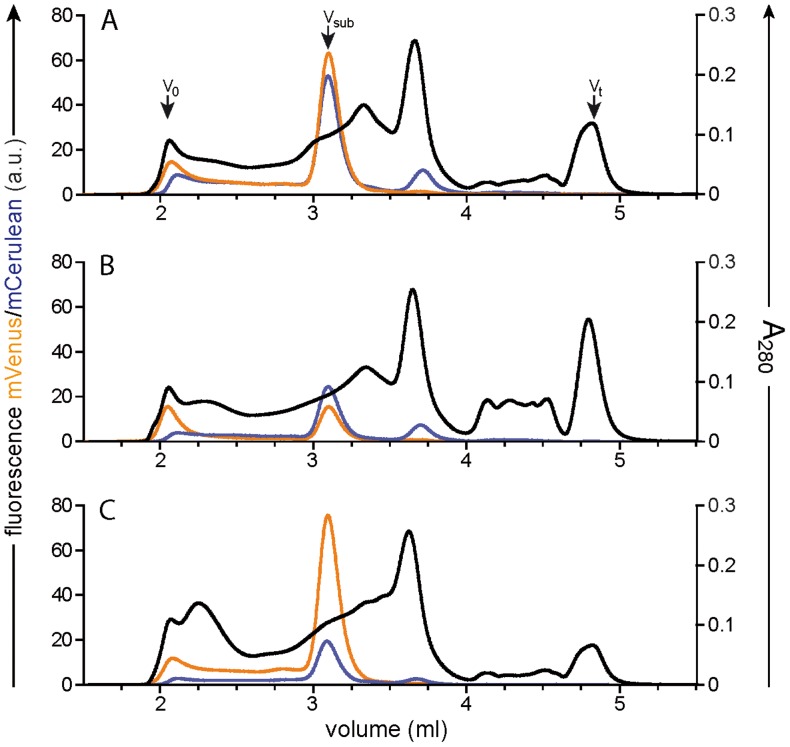
Using MC-FSEC to assess expression levels of individual subunits. Membranes solubilized in *fos*-choline-12 were analysed by MC-FSEC. Each panel (A–C) shows the profile for a sample from an individual colony. Orange indicates mVenus fluorescence, corresponding to TAP1; cyan is the signal for TAP2 from its fused mCerulean protein. The black trace represents absorbance at 280 nm. Arrows mark the void volume of the column (V_o_), total volume (V_t_) and running positions of the TAP subunits (V_sub_).

After membrane preparation and solubilization in the disruptive detergent *fos-*choline-12, MC-FSEC was performed. Figure 3 shows three typical profiles encountered during such an analysis. As the TAP subunits have similar masses, they migrate close to each other, but the overlap seen here is not indicative of heterodimer formation, as previous studies indicate that the complex is unable to withstand solubilisation by this detergent. Two manipulations were performed in order to allow direct comparison. Firstly, the two fluorescence detectors in our system were adjusted to give equal response to equimolar amounts of fluorescent subunits. Isolated mCerulean and mVenus were used for this correction (see Figure S1). Secondly, traces were normalized using the height of the peak at approximately 3.6 ml in the A_280_ absorbance channel. This was found to be more reliable than using protein determination or the whole area of the A_280_ profile. Obviously, those using a different expression system may wish to normalize the total protein content by another method.

In Figure 3A, the almost ideal situation is seen, with high and equal amounts of TAP1 and TAP2 and hardly any aggregated material in the void volume. Panel B is similar except a lower level of expression for both subunits. In contrast, the *Pichia* colony examined in panel C produces a large amount of TAP1 but much less TAP2 (though still reasonable levels). In general, we would prefer a colony such as that analysed in panel A. However, a colony that produces an excess of one subunit over the other, as exemplified in panel C, may still be used as our orthogonal purification allows the isolation of only heterodimeric species.

### Monitoring Multiprotein Complexes in Crude Detergent Extracts

**Figure 4:**
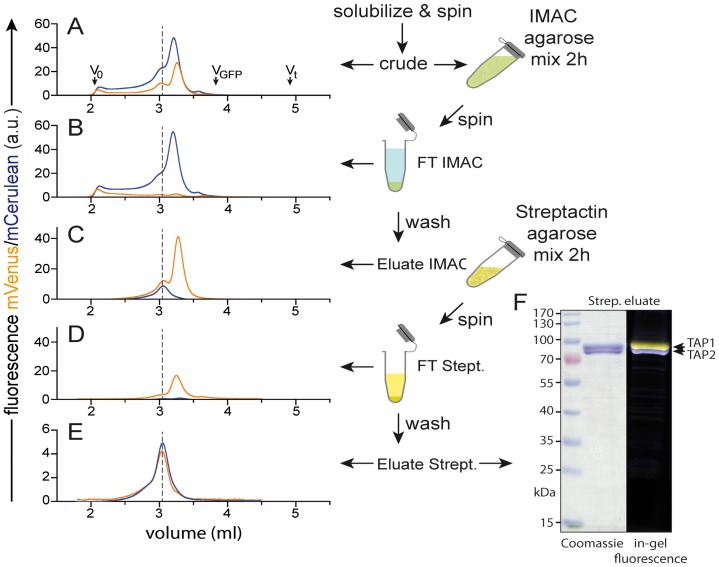
Orthogonal purification of TAP heterodimers analysed by MC-FSEC. (A) Profiles for crude detergent extract of TAP-containing *P. pastoris* membranes. (B) Flow-through of the IMAC resin. (C) Eluate of IMAC resin. (D) Fraction of the IMAC eluate that did not bind to the streptactin beads. (E) Material eluted from the streptactin matrix. Orange traces represent TAP1 (mVenus fluorescence) and cyan profiles are for TAP2 (mCerulean fluorescence). Dashed line indicates the position of the TAP heterodimer, extrapolated from the final streptactin eluate to the crude extract and Ni-NTA eluate. Arrows mark the void volume of the column (V_o_), total volume (V_t_) and running position of free fluorescent proteins (V_GFP_). (F) The final eluate of the orthogonal purification was analysed by SDS-PAGE (Coomassie) and in-gel fluorescence (yellow, TAP1-mVenus and cyan, TAP2-mCerulean).

We next asked whether it would be possible to evaluate candidate solubilization conditions for the purification of a stoichiometrically defined, heterodimeric TAP complex solubilized in mild detergents by simply reviewing MC-FSEC traces of crude extracts. In our experiments we rarely observed free fluorescent proteins in detergent extracts of membranes from *Pichia* cells expressing TAP (Figure 4A). This is probably because free fluorescent proteins are unlikely to be associated with membranes and by routine use of a protease-deficient *Pichia pastoris* strain. However, often several peaks of overlapping TAP1 and TAP2 fluorescence could be seen. Unfortunately, determination of which peak, if any, represents *bona fide* heteromeric TAP complex is not trivial. In a previous study, Kawate and Gouaux were able to resolve the tetrameric P2X receptor from the monomeric form because of the four-fold difference in mass of the two species [25]. Likewise, for larger complexes it may be possible to use MC-FSEC solely for crude detergents extracts. However, in the case of the TAP heterodimer of 150 kDa, the relative difference in mass between detergent-bound monomers and dimers is somewhat less. Thus, we are unable to assess the oligomerization state of TAP and other heterodimeric complexes by analysing crude-detergent extracts alone.

### Rapid Orthogonal Purification of Hetero-oligomeric Complexes

Because we were unable to gain clear information from just crude detergent extracts, we developed an orthogonal purification scheme for rapidly isolating heterodimeric, stoichiometrically well defined complexes. An example using this strategy is shown in Figure 4. The detergent extract, as noted above, shows overlapping peaks of fluorescence from the TAP1 and TAP2 fusion proteins. This material was first incubated with IMAC resin to capture the His_10_-tag on TAP1. All TAP1 subunits bind to the resin with none seen in the unbound fraction. In contrast, most TAP2 remains behind, indicating that it was bound only weakly or not at all to TAP1. This pool is probably monomeric TAP2 but this was not investigated further. After washing and elution of the IMAC beads, a fraction enriched in TAP1 is obtained and two clear peaks are seen on MC-FSEC. In this example, the majority elutes at a position, which does not overlap with TAP2 and therefore does not participate in dimer formation. A smaller amount elutes concomitantly with TAP2 and is presumably in a heterodimer. However, this peak may contain a dimer or oligomer of TAP1 that co-migrates with the TAP heterodimer. To select from these possibilities, the IMAC eluate was adsorbed onto streptactin beads, which bind only the strepII-tag fused to TAP2. As expected, some of the abundant TAP1 peak was observed when the material that did not bind the streptactin-matrix was analysed by MC-FSEC. Specific elution of the streptactin resin by desthiobiotin releases only the heterodimeric complex, as is seen by coelution of the two fluorescent subunits and by the near equal peak heights and areas (Figure 4, bottom). The concentration of TAP in this fraction, calculated by comparing to the signal from pure fluorescent protein is about 125 nM (for a 20 µl injected sample). We have not systematically tested for the lower limit of detection but by increasing detector gain and/or sample volume, 10 nM or less should be detectable. As mentioned above, the two in-line fluorescence detectors were previously adjusted using purified fluorescent proteins so that the same signal intensity corresponded to equimolar amounts of each subunit. By comparing the profiles of the final elution to the crude detergent extract, it is plain that the heterodimer ran as a shoulder of the main peak (see dotted line). In hindsight this might be deduced from the slight shift in position of the subunit signals in the peak at later elution, which indicates that they are monomers. However, in general, ascribing significance to such a small shift would be unwise and undertaking the orthogonal purification strategy allows unambiguous assignment and purification of the stoichiometrically well-defined, heterodimeric complex.

### Effects of Detergent Exchange on Membrane Complexes

It was shown previously using single FSEC that dodecyl-β-D-maltoside (DDM) could be used in the column mobile phase even while testing the influence of multiple other detergents on protein stability [15], [16]. This was because, in these cases, the effects of a deleterious detergent were not reversed during chromatography in the relatively mild detergent DDM. Unfortunately, for the heterodimeric TAP complex DDM cannot be considered mild as isolation in this detergent leads to rapid loss of the ligand-binding activity [17]. Nevertheless, because the MC-FSEC analysis step is rapid (<20 min) we decided to test if it was possible to use a similar strategy in our experiments.

**Figure 5:**
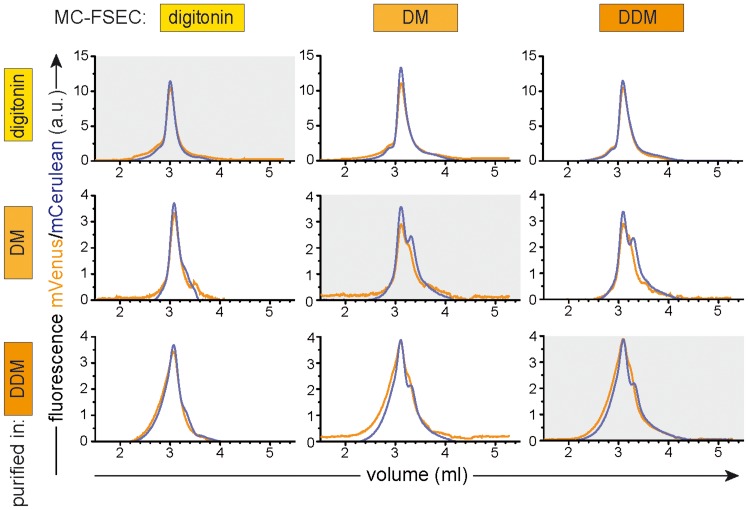
The effect of detergent exchange on membrane complexes. Heterodimeric TAP complexes were purified by orthogonal purification in the three detergents indicated on the left. These samples were then subjected to MC-FSEC in the same three detergents (indicated at the top). For example, TAP heterodimer isolated in digitonin and analysed in DDM is shown in upper right panel, while that purified in the presence of DDM but analysed with the column in DM is shown in lower middle panel. TAP1 subunit is shown in orange traces (mVenus fluorescence) while TAP2 is indicated by cyan profiles (mCerulean fluorescence). Note the small though consistently observed shift for the digitonin-purified sample when run in DM or DDM (compare upper left to upper middle and right panels), which probably is caused by loss of loosely bound detergent or lipid.

We first analysed TAP purified in digitonin (Figure 5, top row). This detergent has been shown previously to be the most suitable for purification of human TAP with full retention of ligand binding, ligand-stimulated ATPase activity, and functional reconstitution in proteoliposomes [17], [24]. When the purified protein was run in decyl-β-D-maltoside (DM) or DDM (top row, middle and right panels, respectively), the peak shape was essentially unchanged except a minor tailing of the peaks. This was encouraging as it suggests that indeed no deleterious effect is occurring during passage through the SEC column.

However, if the protein was purified in DM, followed by MC-FSEC in the same detergent (Figure 5, middle row, middle panel), TAP appears less stable than when purified in digitonin, exhibiting a broad TAP1 peak and a clear, sharp shoulder in the TAP2 trace. A similar profile was observed when DDM was used as column running detergent (Figure 5, middle right). On the other hand, the peak shape of the DM purified TAP was significantly different when the column buffer contained digitonin instead of DM, with TAP1 eluting as a somewhat sharper peak and with a dramatic reduction of the TAP2 shoulder (Figure 5, middle left). The behaviour of TAP purified in DDM is similar, with exposure to digitonin during MC-FSEC apparently converting a broad heterogeneous profile (Figure 5, bottom right) into one less diffuse (Figure 5, bottom left).

Unfortunately, this data suggests that for fragile complexes such as TAP each solubilization/purification detergent must be analysed using the same detergent for column equilibration and elution. Although the changes are relatively small, they are sufficient to potentially mislead. The data also illustrates just how sensitive TAP is to its environment and why it is a difficult-to-handle target.

### Multicolour FSEC for Analysis of Larger Complexes

**Figure 6:**
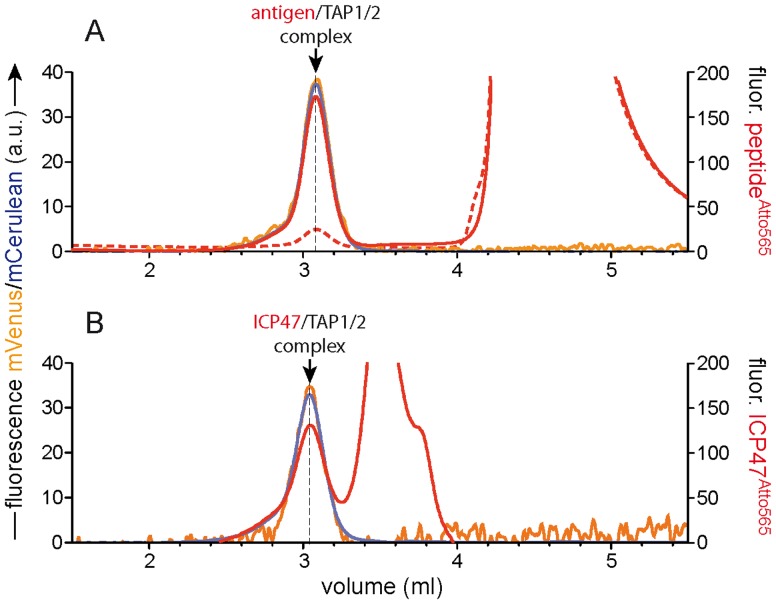
Multicolour FSEC for analysis of larger complexes. (A) Assembly of an antigen/TAP1/TAP2 complex. An antigenic epitope labelled with Atto565 was incubated with purified TAP alone (solid red trace) or with a 100-fold excess of unlabelled peptide before analysis (dashed red trace). The orange line represents TAP1-mVenus subunit, cyan TAP2-mCerulean, and red the Atto565 labelled antigenic epitope. (B) Assembly of an ICP47/TAP1/TAP2 inhibitory complex. The red trace indicates the specific binding of ICP47 labelled with Atto565 (generated by subtraction of traces with a 100-fold excess of unlabelled ICP47). The TAP1-mVenus signal in these experiments shows a high level of noise due to the suboptimal excitation and emission wavelengths used (see Materials and Methods).

The choice of autofluorescent proteins used for tagging TAP1 and TAP2 subunits allows us to use the red range of the visible spectrum for observing interactions of the TAP complex with other proteins or ligands. Figure 6A shows the interaction of purified TAP with an antigenic peptide labelled with Atto565. In the presence of an excess of unlabelled peptide only a small amount of unspecific binding is observed (dashed red trace). As can be seen, the resulting peak corresponding to specifically bound peptide overlaps exactly with TAP1 and TAP2 fluorescence (orange and cyan, respectively). Thus, we are able to monitor both subunits and a ligand bound simultaneously. This is due to a partial overlap of the emission spectra for mVenus and mCerulean fluorescent proteins allowing us to detect both at a common emission wavelength while exciting at two selective wavelengths. This leaves the second fluorescent detector available for one, two or even more colours, provided their spectra can be distinguished (see Figure S1).

As another example, complex formation of the TAP heterodimer and the viral inhibitor ICP47, a 9 kDa protein produced by Herpes simplex virus is shown in Figure 6B. Apart from the inhibitory complex composed of ICP47, TAP1 and TAP2, a second peak of specific binding is apparent, which results from the saturable binding of ICP47 to detergent micelles as reported [26], [27]. This viral inhibitor and other immune evasins are interesting tools as they can arrest TAP in specific conformational states, which could improve stability and aid in crystallization (see review [18]). One can envisage how this approach might allow rapid screening of best conditions for maintenance of TAP activity when combined with thermal denaturation [16].

### Conclusions

FSEC has now been used successfully by several groups to select targets and conditions prior to X-ray crystallography [15], [16], [28]–[31]. Understandably, the method was first applied to monomers or homo-oligomers. However, many interesting and important proteins function as multisubunit complexes. In this report we have shown how one of these membrane multiprotein complexes, the antigen translocation machinery TAP, has been made amenable to this method by extension of the original idea. This approach, which we term multicolour MC-FSEC, acknowledging the earlier method, has allowed us to examine particularly difficult mammalian membrane protein complexes. In establishing the method we decided to use yellow and cyan fluorescent proteins, as their spectra are sufficiently separate to be discriminated. Furthermore, we wish to reserve the longer wavelength domain to employ an additional, for example, red-shifted fluorophores to follow interactions with other proteins as we have demonstrated for binding of an antigenic peptide and a viral inhibitor to TAP.

*Pichia pastoris* suffers from a number of disadvantages as expression host compared to other systems, such as transient transfection of mammalian cells, bacteria, and even *Saccharomyces cerevisiae*. Chief among these is the necessity to select among a number of expressing clones. This is because transformation with expression constructs is reliant on genome integration, which varies widely between cells. The problem is multiplied when multiple genes need to be expressed, *e.g.* to produce a multisubunit complex. However, for TAP *P. pastoris* is the only realistic system for the large-scale protein production required for structural analyses. Both insect cells and *P. pastoris* produce approximately equal amounts of TAP per mg of membrane protein [17], [24], but scale-up of the former systems to produce the levels obtainable from *Pichia* is, for many, cost prohibitive. We therefore have concentrated on this host but believe our approach to be a suitable for any multisubunit assembly, which can be expressed in a suitable host such as *E. coli*, *Lactococcus lactis*, or *Saccharomyces cerevisiae*, where high-throughput platforms are available. In mammalian cells it would be possible by fluorescence activated cell sorting to isolate a pure population of cells expressing multiple fluorescently tagged subunits. Here one could imagine MC-FSEC/orthogonal purification being used to confirm stoichiometric assembly of a complex with functional studies being performed alongside. Likewise, the multiple colours could be used to determine optimal virus titre in an insect cells system.

## Supporting Information

Figure S1 **Fluorescence spectra of fluorescently labelled subunits.**
(TIFF)

Figure S2 **Example of detergent selection using multicolour FSEC.**
(TIFF)
