# Supplementary figures and images for: Multicolour Fluorescence-Detection Size-Exclusion Chromatography for Structural Genomics of Membrane Multiprotein Complexes
Source: PLoS One. 2013 Jun 25;8(6):e67112. doi: 10.1371/journal.pone.0067112 (PMC3692423; doi:10.1371/journal.pone.0067112)

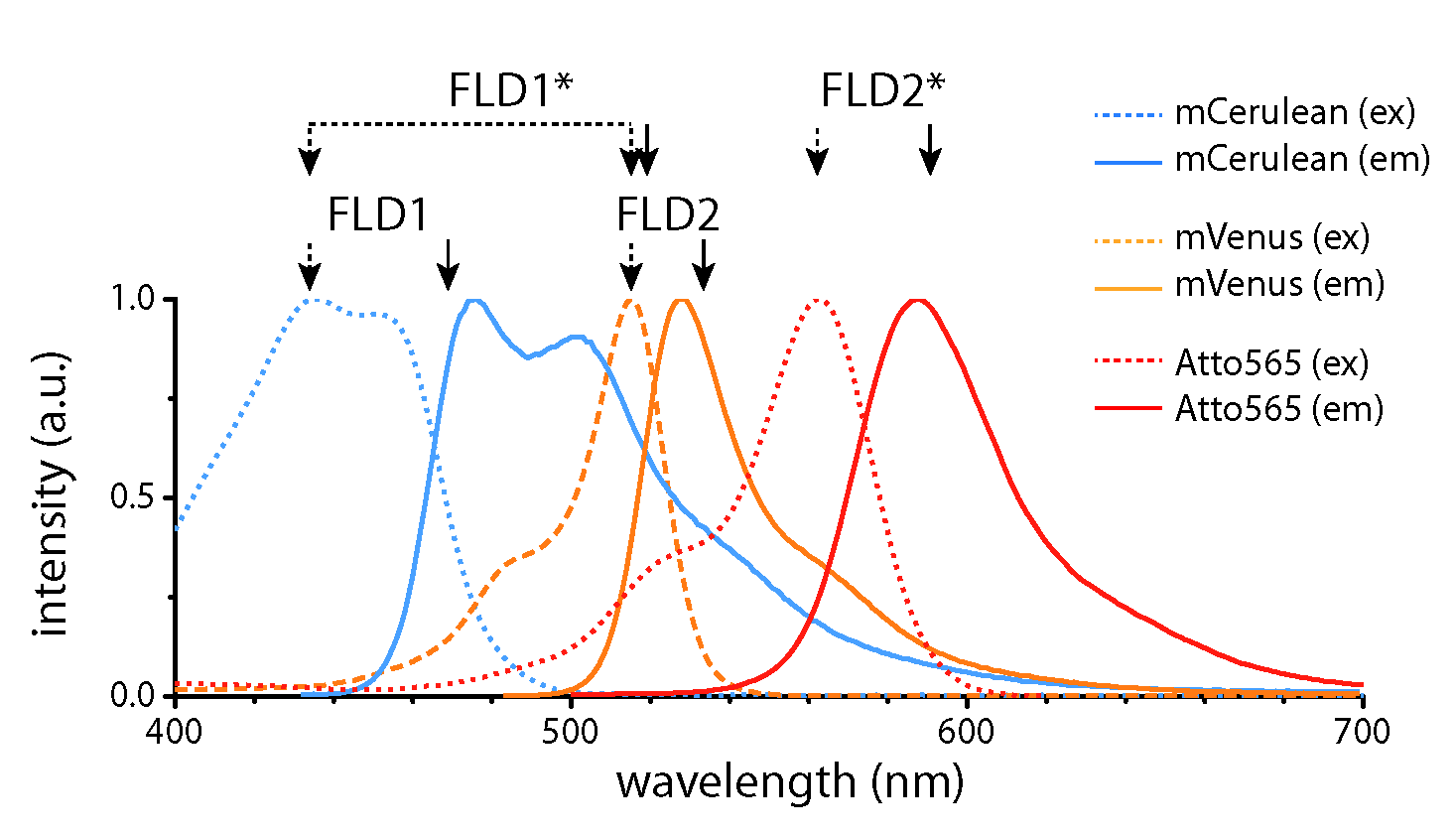

Supplement: Figure S1 — Fluorescence spectra of fluorescently labelled subunits. (TIFF) [file pone.0067112.s001.tiff]

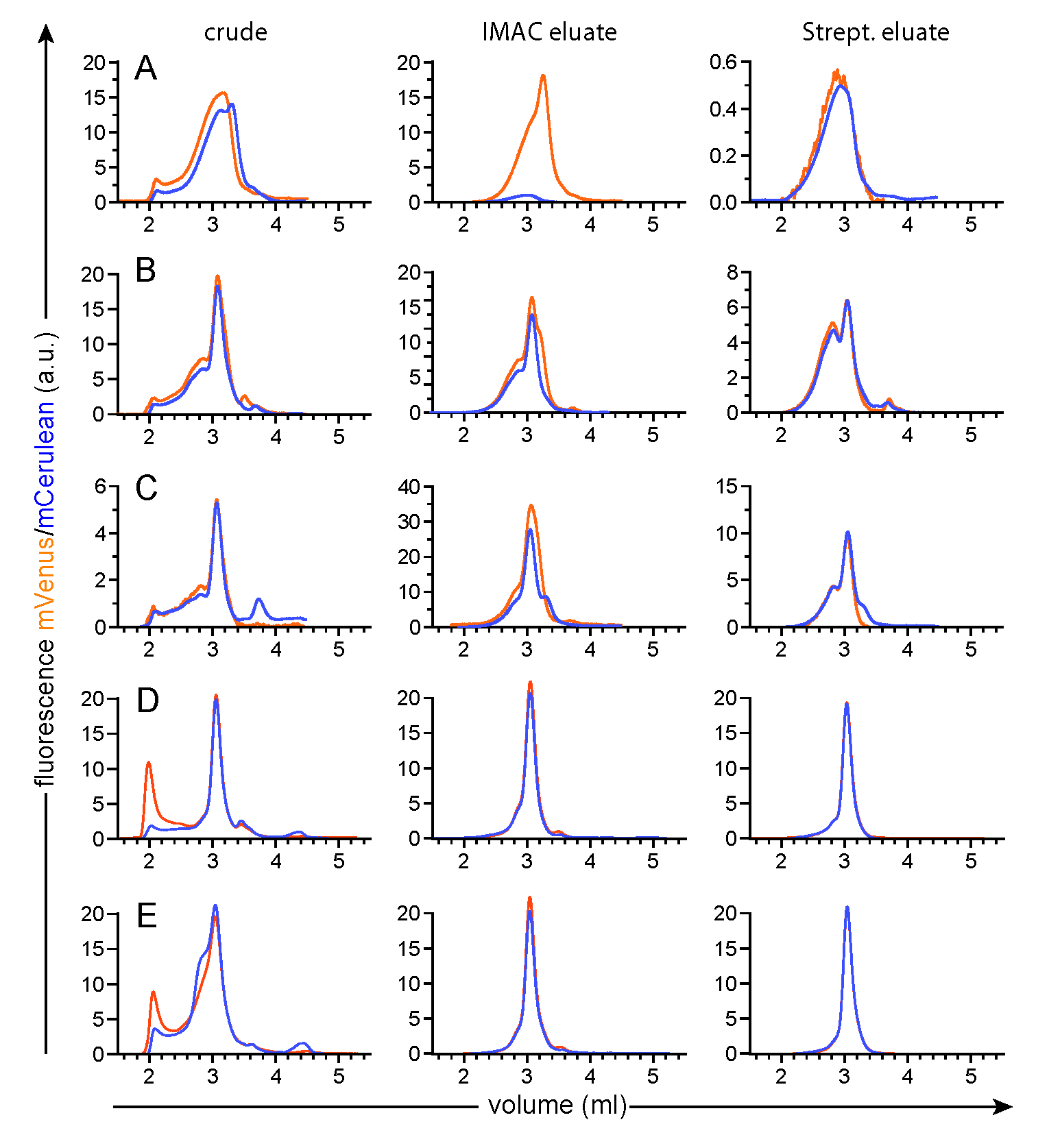

Supplement: Figure S2 — Example of detergent selection using multicolour FSEC. (TIFF) [file pone.0067112.s002.tiff]
